# High-Order Harmonics Generation Using Spherical and Non-Spherical Nanoparticles

**DOI:** 10.3390/nano14121010

**Published:** 2024-06-11

**Authors:** Rashid A. Ganeev, Aigars Atvars

**Affiliations:** 1 Laboratory of Nonlinear Optics, Institute of Astronomy, University of Latvia, Raina Boulevard 19, LV-1586 Riga, Latvia; aigars.atvars@lu.lv; 2 Tashkent Institute of Irrigation and Agricultural Mechanization Engineers, National Research University, Kori Niyoziy 39, Tashkent 100000, Uzbekistan; 3 Department of Optics and Spectroscopy, Voronezh State University, Voronezh 394018, Russia

**Keywords:** high-order harmonic generation, spherical nanoparticles, non-spherical nanoparticles

## Abstract

The conversion efficiency of 800 nm, 65 fs radiation toward high-order harmonic generation (HHG) in laser-induced plasmas containing spherical and non-spherical nanoparticles (NPs) produced during the laser ablation of different metals in water using 1064 nm, 70 ps pulses was analyzed. Non-spherical NPs of different forms (triangle, cubic, bowtie, rod, rectangular, ellipsoid, etc.) were synthesized during the aging of some spherical NPs (In, Al, and Cu) in water. These NPs were then dried on the glass substrates and ablated to produce plasmas comprising nanostructured species of different morphologies. It was shown that harmonic generation in all synthesized non-spherical NPs was less efficient by a factor of at least five than in the initial spherical NP. Meanwhile, the spherical NPs that maintained the morphology state during aging (Ni, Ag, Mn, and Au) showed almost similar HHG conversion efficiency compared to the fresh spherical NPs. In all cases, the HHG conversion efficiency using spherical and non-spherical nanoparticles was notably larger compared to the atomic and ionic single-particle plasmas of the same elemental composition. NP plasmas demonstrated featureless harmonic distributions, contrary to the indium and manganese atomic/ionic plasmas, when the resonance enhancement of harmonics was observed.

## 1. Introduction

The choice of small-sized aggregates for analysis of the frequency conversion of laser radiation toward the extreme ultraviolet (XUV) region using both gas clusters and plasma nanoparticles (NPs) has shown enhancements in harmonic yield with regard to the atomic and ionic species [1,2,3,4]. A larger cross-section of recombination of accelerated electrons and the possibility of combining the electron with the parent multi-particle components through either recombination with the same atom, a neighboring atom, or the multi-particle as a whole were considered the most probable reasons for enhancing the high-order harmonic generation (HHG) yields from such clustered species. Meanwhile, the HHG efficiency in that case can be affected by the spatial shape of NPs. In that case, another process—a variation in quantum confinement-induced growth of the local field in such species—can play an important role in the modification of the nonlinear optical response of these NPs. The inner atoms of such NPs cannot be considered efficient sources of harmonics due to the screening effect of neighboring atoms and the absorption of the laser and harmonic generation. Thus, only surface atoms play a decisive role in the efficient HHG from NPs.

The sizes of spherical NPs (SNPs) vary from a few nanometers to 100 nm. They can be synthesized by different methods (laser ablation in liquids and vacuum, chemical methods, ion bombardment, etc.). Laser ablation of materials allows for the formation of NPs in almost all the solid elements of the periodic table. This method has frequently been used to analyze the low-order nonlinear optical properties of aggregated species [5,6]. In the meantime, the spatial properties of those NPs modify with aging. The NPs can start to aggregate from the very beginning of laser ablation of different materials in liquids (predominantly water). Triangles, bowties, rods, cubes, squares, parallelepipeds, ellipses, and doughnut-like shapes were reported during studies on aged NPs. This process occurs over weeks to months, depending on the features of the initial SNPs and the conditions of their synthesis. 

The low-order nonlinearities like the Kerr effect and nonlinear absorption were compared in the case of the same elemental structures comprising SNPs and non-spherical NPs (NSNPs). The nonlinear absorption measurements showed a distinction in the manifestation of saturable absorption, reverse saturable absorption, and two-photon absorption in the case of different morphologies of NP. Meanwhile, the Kerr-induced nonlinear refractive indices of the suspensions containing SNPs were larger than those of the NSNPs. Notice the absence of similar comparative studies of the HHG in such NPs.

In this study, the generation of the high-order harmonics of a femtosecond laser in plasmas containing NPs with similar elemental composition and different morphologies was analyzed. NSNPs of different forms, such as triangles, cubes, bowties, rods, and ellipses, were synthesized during the aging of some SNPs. These NSNPs were then dried alongside the SNPs on the substrates and ablated in a vacuum chamber to produce the plasmas comprising the species of different structures. The harmonic yields from the plasmas comprising synthesized NSNPs were notably smaller compared to the SNP-containing plasmas.

## 2. Results

### 2.1. Preparation of SNP and NSNP Layers on Glass Substrates

**Figure 1:**
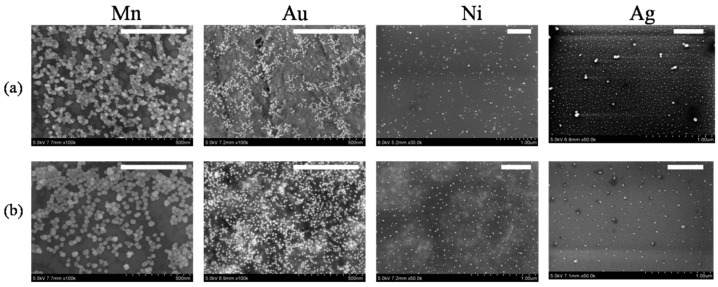
SEM images of four NPs (Mn, Au, Ni, and Ag) synthesized during laser ablation by 70 ps pulses of the metals in water and measured (**a**) one day and (**b**) 30 days from ablation. White lines correspond to 500 nm.

**Figure 2:**
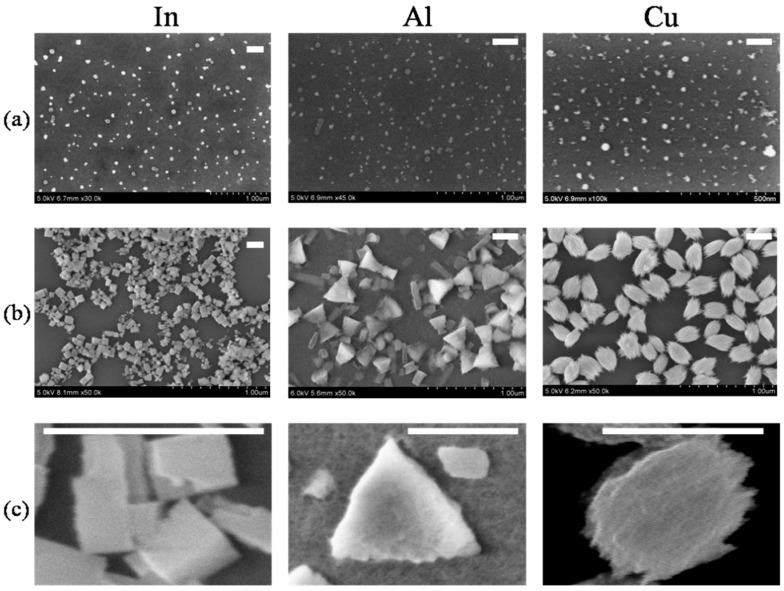
SEM of three NPs (In, Al, and Cu) synthesized during laser ablation by 70 ps pulses of metals in water and measured (**a**) 1 day and (**b**) 30 days from ablation. Panel (**c**) shows the enlarged images of rectangular In NP, triangle Al NP, and elliptical Cu NP. White lines correspond to 200 nm.

The simplicity of NP formation by laser ablation of materials in liquids allowed for the synthesis of NPs of all metals, which were previously used for HHG during the formation of plasmas suitable for efficient frequency conversion of laser pulses toward the XUV region. The procedure for the formation of the SNP and NSNP layers is as follows: Mn, Au, Ni, Ag, In, Al, and Cu were used for ablation and NP formation. The metals were ablated in water using 1064 nm, 70 ps, 10 Hz, 2 mJ pulses from a Nd:YAG laser for 20 min. The prepared suspensions were analyzed using a scanning electron microscope (SEM) to ensure the synthesis of SNPs (Figure 1a and Figure 2a). Then, a part of those suspensions was deposited and dried on glass substrates for the formation of ~0.2 mm thick SNP films. Other parts of the suspension were kept in the refrigerator for a period lasting 30 days, during which the morphology of the NPs was periodically analyzed.

The morphology of Mn, Au, Ni, and Ag NPs remained almost unchanged during the whole measurement period (Figure 1b). The mean sizes of the SNPs were 27 nm (Mn), 12 nm (Au), 10 nm (Ni), and 10 nm (Ag). Studies on the morphology of the NPs at longer periods of aging (up to three months) also did not reveal significant modifications to their structure and size.

Another group of synthesized NPs (In, Al, and Cu) initially also showed a spherical shape in their morphologies (Figure 2a). The mean sizes of the SNPs were 20 nm (In), 18 nm (Al), and 12 nm (Cu). The aging of the SNPs resulted in aggregation towards rectangular (In), triangular (Al), and elliptical (Cu) shapes (Figure 2b). Figure 2c shows enlarged images of the NSNPs. The mean sizes of the NSNPs showed a broader range of aggregated species, with average sizes of ~80 nm (In), ~180 nm (Al), and ~210 nm (Cu).

Aged NPs of Mn, Ni, Ag, and Au, which did not show any modification of their structure, were deposited on glass substrates. Once some of the SNPs (Cu, In, and Al) were transformed during aggregation into complex forms, those suspensions were also used for the formation of 0.2 mm thick films on the glass substrates. Pairs of each metal NP (both initial and aggregated) were used during HHG in the plasmas, comprising SNPs and NSNPs. To ensure maintenance of the NPs’ shapes during ablation of the thin layers of synthesized species in the vacuum chamber, the deposited debris of the plasmas was analyzed in each case of ablation. It was shown that, at the used fluencies of 70 ps, 0.3 mJ heating pulses (*F* = 0.7 J cm^−2^), the morphology of the deposited debris of plasmas was similar to that of the structures of the aged NPs dried on the glass substrates.

### 2.2. HHG Setup

**Figure 3:**
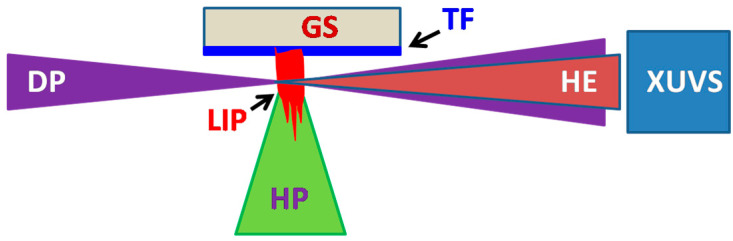
Experimental scheme for harmonic generation in laser-induced plasmas. DP: driving pulses (65 fs, 800 nm); HP: heating pulses (70 ps, 1064 nm); GS: glass substrate; TF: thin films comprising NPs; LIP: laser-induced plasma; HE: harmonic emission; XUVS: XUV spectrometer.

The plasma was formed by a 70 ps, 1064 nm, 10 Hz, 0.7 J cm^−2^ emission focused on the surface of the thin films comprising NPs (Figure 3). These thin films were produced during deposition and drying the NP suspensions on the glass substrate. The conditions of ablation were similar for the SNPs and NSNPs of each material. Thus, one can expect similar plasma densities in those two cases. Each set of measurements was performed on the fresh, non-ablated surface of the films. The bulk metals were also applied for plasma formation to carry out the comparative analysis of HHG from the atomic/ion plasma and NP plasma. The plasma in the former case consisted of atomic (~90%) and singly charged ionic (~10%) metal particles.

After some delay from the beginning of plasma formation (80–400 ns), the driving pulses (65 fs, 800 nm, 10 Hz, 1 mJ) from the femtosecond laser (TSA-10, Spectra-Physics Lasers) were focused inside the laser-induced NP-, atom-, and/or ion-containing plasmas. The delay between heating and driving pulses was accomplished using a digital delay generator, allowing for parameter variation within a broad range (0–10,000 ns). For each case of plasma formation, the optimal delay was maintained, which allowed for maximal harmonic yield generation. The intensity of the driving femtosecond pulses focused inside the laser-induced plasma (LIP) was maintained at *I* = 3 × 10^14^ W cm^−2^.

The plasma and harmonic emissions in the XUV range were analyzed by a hand-made extreme ultraviolet spectrometer (XUVS, Figure 3), which contained a gold-coated cylindrical or spherical mirror and a 1200 grooves/mm flat field grating (FFG, 124, Hitachi Photonics) with variable line spacing. The spectrum was recorded on a micro-channel plate (MCP, F2813-22P, Hamamatsu) with the phosphor screen, which was imaged onto a CCD camera (C4880, Hamamatsu). The movement of the MCP along the focusing plane of FFG allowed for the observation of harmonics in different XUV regions. The fundamental radiation was not blocked after the interaction with the NP plasma but rather entered the XUV spectrometer alongside the harmonic emission. The dispersion of the FFG was sufficient to separate the IR and XUV radiation. The zero-order reflection of the driving radiation (800 nm) from the FFG was blocked inside the spectrometer so that no scattering or stray light was directed toward the MCP. Measurements of harmonic spectra were carried out in a time-integrated mode.

### 2.3. HHG in the Plasmas Containing Spherical NPs

Different cutoffs were observed in the cases of singly atomic and/or ionic LIP and NP LIP. The highest-order harmonics were observed in the former cases, while the notably stronger lower orders of harmonics were obtained in the case of the NP-containing plasmas. This is a frequently reported feature of HHG that appeared when two LIPs (i.e., those that contained mono-particles and multi-particles of the same elemental consistency) were analyzed.

**Figure 4:**
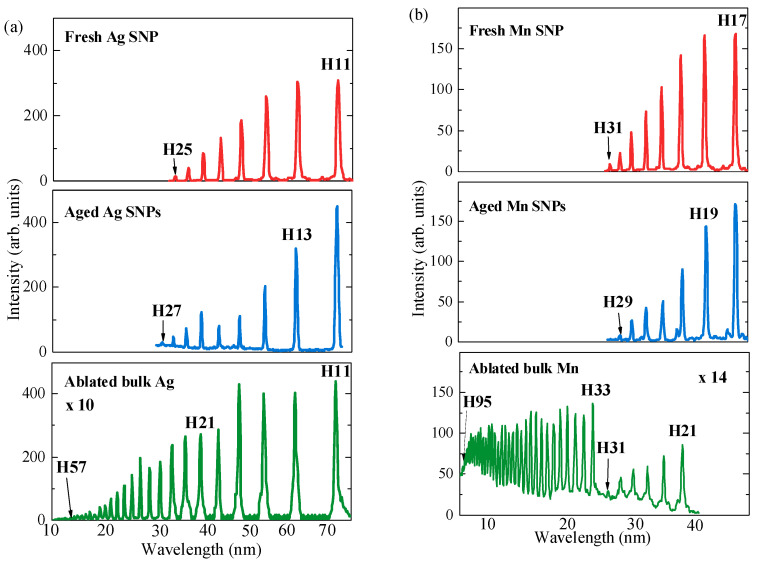
Harmonic spectra from the Ag- and Mn-containing LIP. (**a**) Upper panel: LIP containing Ag SNPs deposited on a glass substrate 1 day after the ablation of silver in the water environment. Middle panel: LIP containing Ag SNPs deposited on a glass substrate 30 days after the ablation of silver in a water environment. Bottom panel: LIP containing Ag atoms and singly charged ions produced during ablation of the bulk silver target in a vacuum chamber. The bottom curve is magnified by a factor of 10 to show the higher orders of harmonics up to the cutoff (H57). (**b**) Upper panel: LIP containing Mn SNPs deposited on a glass substrate 1 day after the ablation of manganese in the water environment. Middle panel: LIP containing Mn SNPs deposited on a glass substrate 30 days after the ablation of manganese in a water environment. Bottom panel: LIP containing Mn atoms and singly charged ions produced during ablation of the bulk manganese target in a vacuum chamber. The bottom curve is magnified by a factor of 14 to show the higher orders of harmonics up to the cutoff (H95).

The upper panel of Figure 4a shows that the lower-order harmonics from the plasma produced on the surface of the film containing Ag SNPs were approximately nine times stronger compared to those produced from the atomic/ionic Ag plasma (bottom panel of Figure 4a; this curve was enhanced by a factor of 10 compared to the upper and middle panels to show the harmonics down to the cutoff region). Optimal ablation conditions were chosen for these two cases, while the driving pulses used remained identical for these two LIPs. The same holds for the other experimental parameters, like the distance between the target surface and the driving beam and the pulse duration of heating pulses.

Localized surface plasmon resonance centered at 400 nm increases the local electrical field around Ag SNPs, which can in principle lead to an effective decrease in ionization potential. According to the three-step model of HHG [7], this decreased ionization potential results in higher conversion efficiency for the low-order part of the HHG plateau and, at the same time, causes a shortening in the harmonic cutoff and plateau range. Additionally, local field enhancement can be attributed to the collective motion of free electrons confined in the narrowly localized regions. In particular, HHG allows for exploiting the local field enhancement induced by plasmons within a metallic nanostructure consisting of bowtie-shaped gold elements on a sapphire substrate [8,9]. HHG resulting from the illumination of plasmonic nanostructures with a short laser pulse from a long-wavelength source was also studied [10]. It was demonstrated that both the confinement of electron motion and the inhomogeneous character of the laser electric field play important roles in the HHG and lead to a significant increase in the harmonic cutoff.

As for the delay between the heating and driving pulses, the optimal values of this parameter notably differed for these two plasmas. The maximal yields from the Ag atoms and Ag SNPs were obtained at 40 ns and 380 ns delays, respectively. The difference in this parameter was caused by different velocities of the particles (atoms, ions, and NPs) spreading out from the target surfaces. An increase in delay above the optimal values led to a gradual decrease in HHG efficiency in both cases. The harmonics were observed with up to 300 ns and 2500 ns delays between the driving and heating pulses for the Ag atoms and ions and Ag SNPs, respectively.

The ratio of the intensities of harmonics generated from the SNP and atomic/ionic plasmas (*I*_SNP_/*I*_at_) strongly decreased with the growth in harmonic order. This ratio was equal to 7.5 in the case of the lower-order 13th harmonic (H13). Meanwhile, the intensity of the cutoff harmonic (H25) generated in the Ag SNP LIP was approximately equal to the one from the Ag atomic/ionic plasma. The harmonics above this order are generated only in the atomic or ionic plasma up to the 57th order (bottom panel of Figure 4a). Thus, application of the Ag SNPs deposited on glass substrate one day after synthesis of the structures during laser ablation of silver in water (“fresh SNP”) demonstrated both a stronger yield in the lower-order harmonics and a low harmonic cutoff compared to the LIP comprising dominantly atomic species. 

For comparison, the harmonic spectra generated in the LIP produced on the surface comprising Ag NPs deposited on glass substrate 30 days from synthesis of the structures during laser ablation of silver in water (“aged SNP”) were analyzed. As was mentioned, the structure of the aged NPs was similar to the SNPs synthesized just after the ablation of Ag in water. The middle panel of Figure 4a shows the harmonic distribution, which is similar to the one presented in the upper panel. The harmonic cutoffs in these two cases were also similar to each other (H27 and H25). These studies show that maintaining the structure of NPs during one-month-long aging resulted in similarities in high-order harmonic yields and cutoffs from the plasmas produced on the surfaces containing the fresh and aged NPs. Thus, these two SNP-containing films being ablated under similar conditions resulted in the predicted equality of the generated coherent XUV radiation characteristics.

The enhancement in low-order harmonics in the case of NP plasma is attributed to the growth observed in a cross-section of the recombined accelerated electron produced during tunnel ionization of the atom on the surface of the NP. This electron can recombine with any atom of the NP with a larger probability compared to the case of a single atom. The lower conversion efficiency in bulk Ag ablation compared to the two studied SNP plasmas could also be explained by the preferable involvement of ions in the harmonic generation in the former case, while, in the case of ablated NP targets, one can expect the appearance of neutral silver clusters alongside the NPs, which may enhance the harmonic emission [11,12]. Correspondingly, the reason for the difference in harmonic emission from the ablated atoms/ions and NPs is attributed to the distinction in the constituencies of plasmas at the optimal conditions of laser ablation for these two cases. Probably, the neutral atoms of silver play a decisive role in harmonic generation from the NP-containing plasma due to the lack of laser-induced ionized NPs. Their involvement in HHG may also explain the low harmonic cutoff observed in the case of NP-containing plasmas.

The maintenance of NPs in the plasma during laser ablation of such structures has a high probability at a low fluence of heating pulses, while their presence and concentration in the plasma volume, where the frequency conversion occurs, are yet to be analyzed directly. The post-ablation conditions of the deposited debris provide indirect information on the nature of those species being presented in LIP, despite the differences between the composition of the plasma formed at its early stages and the deposited material. This difference may arise due to the influence of aggregation on the substrate during the interaction of the heating pulses with the target containing NPs. As was mentioned, the low fluence of heating pulses did not allow for aggregation of the existing NPs but rather served as a condition for the evaporation of the species without modification to their morphology. SEM analysis of debris confirmed a similarity between the initial and deposited Ag NPs, thus pointing out the participation of those nanostructures in HHG. Thus, of morphological analysis of the ablated NPs after their laser-induced deposition on nearby substrates showed that, at moderate laser intensity and the fluence of 70 ps heating pulses on the surface of NP-containing materials (*I* < 2 × 10^10^ W cm^−2^, *F* < 1 × J cm^−2^*)*, the deposited material remained approximately the same as the initial NP.

The application of such samples allowed for the maintenance of stable harmonic emission for a relatively long period of ablation of the target containing NPs in the case of slow movement of the sample used for ablation. This amendment in handling the Ag NP target, in turn, allowed for better-optimized HHG and the achievement of conditions for the generation of higher-order harmonics up to the 27th order (Figure 4a, middle panel).

As was mentioned, the fluence of heating pulses for the Ag SNP targets (0.7 J cm^−2^) was optimized from the point of view of the maximal harmonic yield and taking into account the necessity for maintenance of the intact SNPs in plasma. The effect of heating pulses on HHG from ablated Ag bulk targets was also observed and optimized using the fluence-dependent yield of harmonics. The fluence of heating picosecond pulses in that case was predictably larger (2.5 J cm^−2^) with regard to the optimal ablation of SNP. Maximum harmonic intensity for the ablated Ag bulk target was observed at the energy of heating picosecond pulses of *E* = 2 mJ, with the harmonic cutoff extended up to H57. The harmonic yield and cutoff decreased at *E* > 2 mJ due to the growth in free electron density and the following phase mismatch between the interacting waves. This variation in HHG yield and cutoff confirms that the parameters of heating picosecond pulses strongly affect this nonlinear optical process in an atomic or ionic medium. The optimal laser ablation conditions for the bulk Ag target allowed for the formation of relatively dense plasma, with the electron concentration maintained at ~10% of the plasma concentration. The use of stronger fluence in the heating pulses caused the appearance of a notably larger number of free electrons, which significantly suppressed the conversion efficiency of the harmonics.

Similar features of harmonic emission, as in the case of the Ag SNPs, were observed in the case of Ni, Au, and Mn SNPs, which maintained their structure after one month from the ablation of bulk targets in water. In particular, the manganese fresh NPs deposited on glass substrate one day after the ablation of the bulk Mn target in water allowed for generation up to the 31st order being ablated by the picosecond pulses in the vacuum chamber (upper panel of Figure 4b). The aged SNPs of manganese showed similar harmonic characteristics (cutoff and harmonic yield; see middle panel of Figure 4b).

Manganese plasma allows for the generation of the harmonics of 800 nm class lasers above the 100th order. Another interesting peculiarity of HHG in this plasma created on the surface of bulk manganese is a resonance enhancement in the 33rd harmonic. The harmonics above this order resemble the second plateau after the first one. The properties of this plasma were examined in a few studies and demonstrated, particularly, a strong H33, especially in the case of few-cycle driving pulses [13]. Earlier, it was shown that the enhancement in single harmonics in Mn plasma with harmonic energy near the energy of autoionizing states in manganese ions can be interpreted in terms of the usual three-step scenario of HHG [14]. In particular, the main features observed during HHG from the plasma produced by laser ablation of the bulk Mn target were successfully reproduced during those calculations. For both 800 and 400 nm wavelengths, these features are caused by atomic structure effects in the radiative recombination cross-sections of Mn III ions (or, equivalently, in the photoionization cross-sections of Mn II singly charged ions). Analysis of these microprocesses is crucial for understanding the observed peculiarities of plasma harmonic spectra.

The harmonic spectrum from the LIP produced on the surface of the Mn bulk target using 800 nm radiation is shown in the bottom panel of Figure 4b. Harmonics up to H95 were observed in the present studies using the optimal ablation of the Mn target by 70 ps pulses. The intensity of the second plateau-like distribution of the highest-order harmonics was stronger than that of the first plateau shown in the right part of the same panel. The strong 33rd harmonic (*E*_ph_ ≈ 51 eV) significantly exceeded the nearby lower-order harmonics (H21–H31). As in all cases of the comparison of harmonic yields from the atomic and NP plasmas, the latter medium allowed for the generation of significantly stronger lower-order harmonics (upper and middle panels of Figure 4b). The harmonics from the atomic/ionic Mn plasma (bottom panel) were presented with a 14-fold enhancement factor to compare with the upper and middle panels.

Earlier, the theoretical study of HHG from transition metal elements Mn and Mn^+^ using full-dimensional, all-electron, first-principles simulations was reported [15]. The HHG spectra calculated with the time-dependent complete active space self-consistent field and occupation-restricted multiple active space methods exhibited a prominent peak in the *E*_ph_ ≈ 50 eV region. Artificially freezing 3*p* orbitals in simulations resulted in their disappearance, which showed the essential role played by 3*p* electrons in the resonant harmonics. Further transition-resolved analysis unambiguously identified constructively interfering 3*p*-3*d* giant resonance transitions as the origin of the resonant harmonic, as also implied by its position in the spectra.

A comparison of the HHG spectra from the atomic/ionic Mn plasma (bottom panel) and SNP plasma (upper and middle panels) demonstrated a significant modification of harmonic distribution in the latter cases. One can realize the disappearance of the resonance-enhanced process resulting in the generation of strong harmonics starting from H33 in the case of the SNP plasmas produced during ablation of the fresh and aged Mn NPs.

Thus, the difference in harmonic distribution in the case of Mn-containing plasmas is attributed to the involvement of different constituents, neutrals, and singly charged ions. In the case of NPs, the neutral atoms on the surface of this aggregate participate in HHG, thus restricting the harmonic cutoff. Contrary to that, in the case of the plasma produced on the surface of solid Mn, some of the ejected material becomes presented in an ionized state. The harmonics originating from Mn II ions significantly depend on the strong ionic transitions possessing large oscillator strengths and can show the resonance enhancement in single harmonics and nearby higher-order harmonics. This resonant amplification of a high-order harmonic was earlier explained by the influence of the transitions of single charged metal ions. This process was not observed during our experiments with Mn SNPs. Meanwhile, one can assume that the disintegration of Mn-containing NPs at high heating fluence can produce ions in the plasma area and restore the resonant amplification in harmonics.

### 2.4. HHG in the Plasmas Containing Non-Spherical NPs

**Figure 5:**
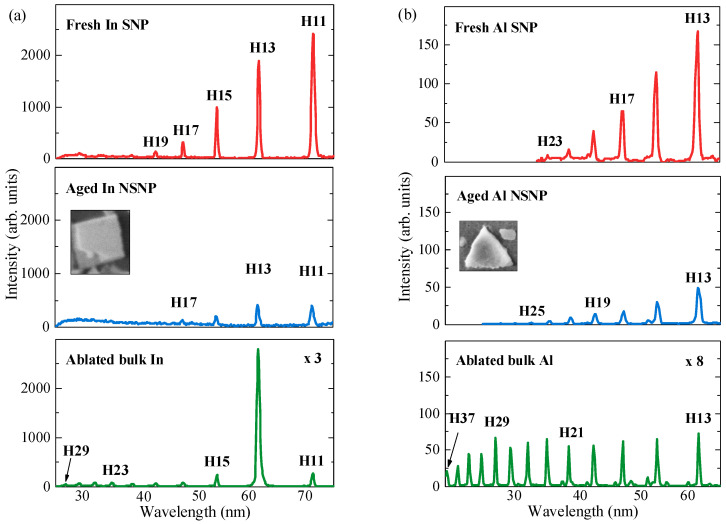
Harmonic spectra from the In- and Al-containing LIPs. (**a**) Upper panel: LIP containing ablated indium SNPs deposited on glass substrate 1 day after ablation of indium in a water environment. Middle panel: LIP containing ablated indium NSNPs deposited on glass substrate 30 days after ablation of indium in a water environment. The inset shows the image of a single rectangular In NSNP. Bottom panel: In plasma produced during the ablation of bulk indium target in the vacuum chamber. The bottom curve is magnified by a factor of 3 to show the higher-order harmonics. The harmonic cutoff in this case is H39 (not shown in this graph). (**b**) Upper panel: LIP containing the ablated aluminum SNPs deposited on glass substrate 1 day after ablation of aluminum in a water environment. Middle panel: LIP containing ablated aluminum NSNPs deposited on glass substrate 30 days after ablation of aluminum in a water environment. The inset shows the image of a single triangular Al NSNP. Bottom panel: Al plasma produced during ablation of the bulk aluminum target in the vacuum chamber. The bottom curve is magnified by a factor of 8 to show the higher orders of harmonics. The observed harmonic cutoff in this case is H47.

Similar HHG experiments were carried out using the fresh SNPs and aged NSNPs produced during laser ablation of In, Cu, and Al in water (Figure 2a,b), as well as the LIP containing atoms and ions of ablated solid metals. The procedure for ablation of the thin films comprising the fresh and aged NPs of these metals was identical to those described in the previous subsection. The upper panel of Figure 5a shows the harmonic spectrum from the fresh spherical NP of indium. The featureless decaying harmonics between the 11th and 21st orders resemble those obtained in the case of the Ag and Mn fresh SNPs (Figure 4, two upper panels). Meanwhile, the application of the film containing aged non-spherical In NPs for ablation and HHG resulted in a notable (sixfold) drop in conversion efficiency (Figure 5a, middle panel) compared to the In SNP plasma. In that case, the plasma contained dominantly large rectangular NP species—cubes and rods (see inset to the middle panel). In both cases, the harmonic cutoff did not exceed H19. The participation of neutral indium atoms in HHG was assumed during these experiments, analogously to those described for the Ag and Mn NPs. As one can see, the application of the NSNPs resulted in a smaller harmonic yield compared to that of the SNPs. 

The application of the LIP produced on the surface of bulk indium for harmonic generation led to drastic changes in the harmonic distribution (Figure 5a, bottom panel). The harmonic cutoff was extended up to the 39th order (not shown in this curve). The most important peculiarity here was a significant enhancement in the 13th harmonic over other neighboring harmonics. H13 was ~12 times stronger than the 11th and 15th harmonics. This enhancement of a single harmonic in the vicinity of 62 nm (*E*_ph_ ≈ 20 eV) was reported in previous studies of In LIP using different driving pulses, starting from the first observation of this resonance-enhanced process [16]. Particularly, the effect of laser chirp on this harmonic generated in an indium ablation plume by few-cycle 775 nm pulses was analyzed, and it was found that the resonant emission showed strong chirp dependence, with pronounced modulations and an asymmetry with regard to the chirp sign [17]. The simulations qualitatively reproduced the main features of the experiment, particularly the positive/negative chirp asymmetry and modulated resonant emission structure as a function of group delay dispersion.

This feature was observed only in the ion-containing In plasma. The application of neutral plasmas or plasmas containing the molecular In-comprising species did not result in the generation of a strong single harmonic in the vicinity of 62.2 nm. One can conclude that the emission spectrum of harmonics in the range of 25–75 nm (bottom panel of Figure 5a) studied in the present paper originated predominantly from the singly charged indium ions. The NP-containing indium plasma, comprising either spherical or rectangular aggregates, caused the harmonic generation from the neutral atoms located on the surface of these aggregates. Correspondingly, no resonance effect was observed at the used fluencies of the heating pulses in this case. Some signs of resonance enhancement of H13 in NP plasmas appeared only in the case of very strong ablation of the films containing In NP (3 J cm^−2^). In that case, the NPs disintegrated into clusters and atoms. The latter species were partially ionized at these conditions, resulting in the interaction of the driving femtosecond pulses with the ions of indium. However, this process was also followed by a very strong incoherent emission of the plasma plume, which almost suppressed the harmonic emission.

The harmonic spectrum shown in the bottom panel was increased by a factor of three compared to the two upper panels to demonstrate the comparison between the emissions from the atomic/ionic plasma and NP plasmas. One can see that all lower-order harmonics, except H13, from the atomic/ionic plasma (bottom panel) were 25 (H11) to 6 (H19) times weaker compared to the harmonics generated in the plasma comprising SNPs (upper panel). The only harmonic that showed a stronger or comparable yield with those from the SNP and NSNP plasmas was that of the 13th order. Thus, one can conclude that the resonance-enhanced process of harmonic generation is comparable with the nanoparticle-enhanced process during HHG in some plasmas.

The next plasma, which comprised NSNPs, contained triangular aluminum NPs. It showed similar harmonic generation properties (Figure 5b, middle panel) to the one comprising rectangular In NPs (Figure 5a, middle panel). The inset to this figure shows the shape of the used Al NSNPs for HHG. In both cases (Al SNP and Al NSNP), the harmonic cutoffs were approximately the same (H23 and H25, respectively; compare the upper and middle panels of Figure 5b). Again, like for the indium NPs, a notable (fivefold) drop in conversion efficiency in the lower-order harmonics was observed in the case of the Al NSNP LIP compared to the Al SNP LIP.

Contrary to that, the Al atoms/ions containing plasma demonstrated a significantly larger cutoff (H47, not shown in the bottom panel of Figure 5b). The featureless plateau-like shape of harmonic distribution along the whole range of emission pointed out the origin of harmonics from the ionic species. The ratio of H13 generated from the SNP plasma and atomic/ionic plasma was equal to 18, while this ratio dropped to 5 in the case of the Al NSNPs. Similar features were observed in the cases of Cu SNPs, Cu NSNPs, and Cu atomic/ionic plasmas.

One can observe some weak spikes at around H13 to H21 (bottom panel of Figure 5b) in the case of HHG in atomic/ionic Al produced during bulk target ablation. These are the second orders of diffraction from the grating in the case of strong higher-order harmonics (H27 to H43).

## 3. Discussion

The variation in NP morphology led to a decrease in HHG conversion efficiency for the large non-spherical species. This process could be related to a decrease in the local field enhancement in the vicinity of the larger species. Particularly in the case of indium NPs, the variation in the absorbance of NSNP suspension with regard to SNP suspension in the UV range showed a disappearance of the surface plasmon resonance (SPR) in the former case. This peak (262 nm) has previously been reported, and it was shown that its position depends on the mean size of the spherical NPs and surrounding liquid [18,19]. The same can be said about the presence and disappearance (or suppression) of the SPR (*λ* ≈ 650 nm) in the case of Cu SNPs and NSNPs, as well as in the case of Al SNPs and NSNPs (SPR *λ* ≈ 240 nm), respectively. Notice that the formation of Cu NSNPs was followed by the appearance of an absorption peak at *λ* ≈ 300 nm and the disappearance of the peak at *λ* ≈ 650 nm, similar to earlier reported modifications of the absorption spectrum of ablated Cu species [20].

Contrary to the modifications in the absorption spectra of In, Cu, and Al fresh SNP and aged NSNP suspensions, silver NPs did not show any variation in their SPR (~405 nm) during the whole range of the aging of their suspensions. The same feature was observed in the case of the SPR of Au SNPs. The absorption peak associated with those NPs remained almost unchanged (527 nm and 537 nm) during the whole range of their measurements. The absorption spectra of Ni NP suspensions in both cases (before and after aging) showed an increase in absorbance close to 200 nm, alongside a very broad maximum centered at *λ* ≈ 410 nm, which was previously associated with the SPR of NiO NPs [21,22]. Also, Ni SPRs at 206 nm and 376 nm were determined. Meanwhile, Mn suspensions did not demonstrate SPR peaks, neither in fresh SNP suspensions nor after the long aging of the suspensions. Probably, the weak SPR was in the UV range, which cannot be precisely distinguished by the spectroscopic facility used during the present study. Previously, the SPR of manganese NPs was analyzed [23]. In the absorption spectrum at 300 nm (4.13 eV), the band attributed to SPR in Mn nanoparticles was reported.

Thus, the disappearance of the SPR in the case of NSNPs highlights the suppression of the local field-induced enhancement in the nonlinear optical response of these media, particularly leading to reduced conversion efficiency toward the high-order harmonics.

The plasmonic field enhancement in harmonics can be realized when plasmonic nanostructures are illuminated by an intense laser and have a particular spatial dependency, depending on the geometrical shape of the nanostructure. One can expect that the strong non-homogeneous character of the laser-enhanced field plays an important role in HHG and significantly extends the harmonic cutoff. As was mentioned, previously, application of the plasmon effect resulted in an enhancement in HHG yield in the gases [8,9,10]. This enhancement was achieved by exploiting the local field enhancement induced by resonant plasmons within a metallic nanostructure consisting of bowtie-shaped gold elements on a sapphire substrate. The enhancement factor exceeded 20 dB, which was sufficient to produce XUV wavelengths down to 47 nm by injection with an argon gas jet. However, this process was observed in the case of propagation of the driving beam along a set of spatially fixed triangles so that the local fields of the corners of those triangles were properly aligned in the focal area of the laser beam. Contrary to this configuration of the corners of triangles, in the present case, the triangle-shaped Al NPs were chaotically distributed in the plasma, thus canceling the additive influence of the local fields introduced by each of these NPs on the harmonic generation.

Further, though the average concentrations of the NPs of different morphologies and similar elemental consistency were approximately equal to each other, the number of atoms in those structures (small SNP and large NSNP) directly participating in HHG differed from each other. In both cases, the atoms on the surfaces of the NPs were the main participants in the frequency conversion of laser radiation. The atoms placed at a small distance from the surfaces of the NPs could also generate harmonics. Meanwhile, the other inner atoms of the SNPs and NSNPs did not generate harmonics due to a lesser laser field or stronger shielding of the NP field, which prevented the tunneling electrons from further acceleration and recombination with the atoms of these multi-particle structures. Correspondingly, a large part of the atoms of the NPs do not participate in HHG. Since the ratio of the inner atoms to surface atoms in the case of modified aggregated NPs (cubes, rods, triangles, ellipses, etc.) is larger compared with the same parameter in the case of small initial spherical NPs, a smaller number of emitters participate in harmonic generation in the case of NSNPs. Correspondingly, the HHG conversion efficiency in the case of NSNPs will be predictably smaller compared to the harmonic generation by SNPs.

It is important to completely remove the HHG produced from the particles, which could be ablated from the glass substrate. We used a clear glass surface to ablate it at the fluence of the heating pulses, as in the case of the ablation of the thin films containing NPs of different morphologies, and did not observe harmonic generation. Moreover, the application of significantly larger fluencies of the heating pulses also did not result in the ablation of glass substrates due to the extremely small absorption of the surface at the wavelength of laser radiation used. Thus, the observed harmonics are generated exclusively from the NPs of different morphologies.

The prevalent characteristic of nanoparticles is their high surface area-to-volume ratio. Specifically, spherical nanoparticles exhibit the greatest surface area-to-volume ratio among all nanoparticle morphologies. Consequently, spherical nanoparticles demonstrate superior HHG properties compared to non-spherical particles. The findings presented in this manuscript align with this assumption. The novelty of these studies lies in their comprehensive description of the results obtained from experimental data showing the direct comparison of the harmonic yield from two groups of NPs. Thus, the significance of conducting HHG measurements on aged particles compared to freshly prepared spherical samples is related to the determination of the prevailing role of the surface area-to-volume ratio in HHG for different morphologies of the synthesized NPs. Correspondingly, studies on different durations of aging will provide significance for the selection of suitable target materials for building high-intensity harmonic sources.

Notice that the SEM images do not provide a clear indication that the non-spherical particles are homogeneous in nature. This heterogeneity is seen in the case of the aged Al NPs when, alongside the triangle form, the rod and rectangular forms appear after 1 month of aging (central panel of Figure 2b). Even triangular NPs exhibit irregularity in size. It is essential that the heterogeneity in shape and size does not cancel the conclusion that the spherical NPs allow for stronger HHG generation. The relatively homogeneous suspensions of rectangular In NPs and elliptical Cu NPs (left and right panels of Figure 2b) showed a tendency to decrease HHG yield, similar to less homogeneous triangular Al NPs. Therefore, it is clear that the uniformity of the non-spherical particles does not influence the conclusion about the prevailing role of spherical form over other forms of NPs in the generation of larger harmonic yields.

These studies were carried out with several of repetitive processes and systematic studies. From a scientific point of view, such a systematic study is useful, as it provides a good reference. In particular, it might help during studies on HHG mechanisms in different NPs.

The inactive inner atoms of NPs, which do not participate in HHG in the case of NSNPs, represent a significant percentage of the whole set of particles in the laser-induced plasma. It is difficult to estimate the percentage of atoms from the whole number of NSNPs of different shapes that can be considered active. In other words, it is almost impossible to define how many atoms lying beneath the surface of NSNPs can be considered as active sources of harmonics. Their role diminished with distance from the surface of the NPs. The following question may arise: How can this effect be included in the comparative measurement of conversion efficiency when using SNPs and NSNPs? The quantitative measurements of the harmonic spectra at the optimal ablation conditions of the spherical and non-spherical NPs included the maintenance of the same collection time of XUV spectra, with further determination of the line-outs of harmonic emission in different ranges of XUV. The harmonic yield was measured using a CCD camera and directly depended on the available amount of converting particles (i.e., the outer and near-to-surface atoms, which can be ionized by the tunnel effect, and then the electrons can be accelerated in free space). Another issue is the behavior of the free electrons produced by tunnel ionization beneath the surfaces of the NSNPs. They can be partially or entirely shielded by the neighboring atoms of the NSNPs prior to moving to free space to accelerate and recombine with any atom on the surface.

The discussion related to the influence of the morphology of the aggregated NPs is important for understanding the mechanisms of HHG enhancement. During these studies, NPs of various shapes were synthesized and aggregated in the case of some ablated metals. For each of the ablated metals, particularly indium, aluminum, and copper, the modification of the shape of the NPs after aging occurs differently. Determining a process for making NPs of similar shapes is a challenging task. The aged NPs in the case of In, Al, and Cu ablation represented the majority of species, such as rectangles, triangles, and ellipses, respectively. The percentage of NSNPs in the aged suspensions varied depending on the ablated material. Particularly in the case of aged Cu NPs, almost all (i.e., more than 95%; see Figure 2b for copper) aggregated NPs had an elliptical shape. The raw estimates for rectangular In NSNPs (~80%) and triangle Al NSNPs (~70%) were deduced from the analysis of the SEMs analogous to those shown in Figure 2b for these metals. Notice that in the case of aged Al NP suspensions, we also observed rod-like species (see Figure 2b for aluminum). It was difficult to precisely determine the percentage of non-aggregated SNPs in these three cases. Actually, as one can see (Figure 2b), there are almost no spherical NPs in the aged suspensions of those metals (compare with Figure 2a).

The important issue here is the maintenance of the shape of non-spherical NPs during the ablation of thin films containing those species and the formation of plasma for HHG. This condition is a requirement for the judgment that harmonic generation occurs from the non-spherical NPs rather than from the disintegrated components of these species. To confirm the presence of NSNPs in the plasma, we analyzed the debris of ablation deposited on the nearby substrates using the SEM technique. These SEM images showed that the debris represented the same morphological shapes as those measured prior to the ablation in the vacuum chamber. The same analysis was performed in the case of ablation of the spherical NPs during HHG from these species. Thus, we can judge that the harmonics were generated dominantly from the NSNPs and SNPs in these two cases.

## 4. Conclusions

We analyzed a difference in the conversion efficiency of 800 nm radiation toward the high-order harmonics in laser-induced plasmas comprising spherical and non-spherical NPs produced during the laser ablation of different metals in water. Non-spherical NPs of different forms (triangular, cubic, rectangular, bowtie, rods, etc.) were synthesized during the aging of some spherical NPs (In, Al, and Cu). It was shown that harmonic generation in all synthesized non-spherical NPs was less efficient than in the initial spherical NPs. In all cases, the HHG conversion efficiency in the spherical and non-spherical NPs was notably greater compared to the atomic and ionic single-particle plasmas of the same elemental consistency. The reasons for decreasing the conversion efficiency toward the harmonics in the case of NSNPs compared to SNPs were discussed.

Our studies show that the significance of conducting HHG measurements on aged particles compared to freshly prepared spherical samples is related to the determination of the prevailing role of the surface area-to-volume ratio in HHG for different morphologies of the synthesized NPs. Correspondingly, studies on different aged small-sized species can provide information for the selection of suitable target materials to build high-intensity harmonic sources.

Since the ratio of the inner atoms to surface atoms in the case of modified aggregated NPs (cubes, rods, triangles, ellipses, etc.) is larger compared with the same parameter in the case of small initial spherical NPs, a smaller number of emitters participate in harmonic generation in the case of NSNPs. Correspondingly, the HHG conversion efficiency in the case of NSNPs will be predictably smaller compared to the harmonic generation using SNPs. The important issue here is the behavior of free electrons produced by tunnel ionization. They can be partially or entirely shielded by the neighboring atoms of NSNPs prior to moving to free space to accelerate and recombine with any atom on the surface.

## Data Availability

The datasets used and analyzed during the current study are available from the corresponding author upon reasonable request.
